# Integration of single cell and bulk transcriptomic analyses identifies FAM189A2 as a key prognostic gene in lung cancer

**DOI:** 10.3389/fimmu.2025.1701806

**Published:** 2026-01-06

**Authors:** Peng Guo, Mengqi Xiang, Jing Chen, Huachuan Zhang

**Affiliations:** 1 Department of Pathology, Sichuan Clinical Research Center for Cancer, Sichuan Cancer Hospital & Institute, Sichuan Cancer Center, Affiliated Cancer Hospital of University of Electronic Science and Technology of China, Chengdu, China; 2 Department of Medical Oncology, Sichuan Clinical Research Center for Cancer, Sichuan Cancer Hospital & Institute, Sichuan Cancer Center, Affiliated Cancer Hospital of University of Electronic Science and Technology of China, Chengdu, China; 3 Department of Thoracic Surgery, Sichuan Clinical Research Center for Cancer, Sichuan Cancer Hospital & Institute, Sichuan Cancer Center, Affiliated Cancer Hospital of University of Electronic Science and Technology of China, Chengdu, China

**Keywords:** FAM189A2, lung cancer, prognostic signature, risk model, scRNA-seq, tumor microenvironment

## Abstract

**Background:**

Lung cancer remains the leading cause of cancer−related mortality. Single−cell RNA sequencing (scRNA−seq) enables high−resolution mapping of tumor microenvironment, but how malignant cell states connect to patient outcomes and therapeutic vulnerabilities is not fully understood.

**Methods:**

We analyzed scRNA−seq profiles from 13 primary lung tumors and matched normal lung tissues (48,149 cells). Malignant versus non−malignant epithelial cells were defined by inferCNV, co−expression programs resolved with Hotspot, transcription factor regulons inferred by pySCENIC, and differentiation potential estimated by CytoTRACE and diffusion pseudotime. Key malignant programs were cross−checked in an independent LUAD scRNA−seq cohort (GSE131907). Program signatures were projected into TCGA−LUAD to build an eight−gene LASSO−Cox risk model, which was further validated in two external NSCLC/LUAD cohorts (GSE30219 and GSE31210). Immune contexture was inferred by CIBERSORT and TIDE, drug sensitivities predicted with pRRophetic, and the function of FAM189A2 tested by knockdown and overexpression in A549 and NCI−H23 cells.

**Results:**

We delineated 12 major cell populations and six malignant epithelial subclusters, including an early GPRC5A^+^ subpopulation with high developmental potential. Malignant programs corresponding to cell−cycle and inflammatory states (Modules 8, 14 and 16) were associated with worse overall survival, whereas Module 15, preferentially active in the GPRC5A^+^ state, was linked to better outcomes. An eight−gene signature (LAMA5, ACTB, B4GALT1, KLF5, KRT18, FAM189A2, SLC34A2, S100A11) robustly stratified patients into high− and low−risk groups across TCGA−LUAD and two validation cohorts. High−risk tumors displayed an immune−enriched yet matrix−restrained microenvironment with higher CD8^+^ T cells, whereas low−risk tumors showed greater predicted sensitivity to Aurora kinase, IGF−1R, mTOR and TGF−β inhibition. FAM189A2 was down−regulated in tumors, higher expression predicted better survival, and bidirectional perturbation in A549 and H23 cells demonstrated that loss of FAM189A2 enhanced, while overexpression attenuated, migration and invasion *in vitro*.

**Conclusions:**

Integrating single−cell and bulk transcriptomes links malignant epithelial state programs to prognosis, yields a practical eight−gene risk model validated in multiple LUAD cohorts, and nominates FAM189A2 as a putative tumor suppressor and potential biomarker in lung cancer. These findings suggest testable strategies for risk stratification and therapy selection that warrant prospective evaluation.

## 1 Introduction

Lung cancer is the leading cause of cancer−related mortality worldwide (1). Although the adoption of molecularly targeted agents and immune−checkpoint inhibitors has improved outcomes for selected subgroups, the overall 5−year survival rate for lung cancer still hovers below 20 % (2, 3). Two factors largely account for this shortfall. First, most patients present with advanced or metastatic disease, precluding curative surgery (4). Second, current systemic therapies benefit only those tumors that harbor specific driver alterations or an inflamed immune milieu, and even in these cases resistance inevitably develops (5, 6). Pinpointing new molecular vulnerabilities therefore remains a high priority.

scRNA−seq provides a powerful means to chart the cellular landscape of tumors at unprecedented resolution. By capturing transcriptomes one cell at a time, scRNA−seq distinguishes rare populations and transitional states that bulk RNA analyses obscure, shedding light on mechanisms of tumor evolution, immune evasion and drug resistance. Recent scRNA−seq studies in lung cancer have delineated immune−cell infiltration patterns, mutation−specific transcriptional programs and early premalignant niches (7–9). Yet most of these efforts have centered on a limited number of molecular subtypes or immune components.

Here, we analyzed single−cell transcriptomes from 13 primary lung cancer and matched normal lung tissue. Using harmony−integrated clustering, inferCNV, Hotspot and CytoTRACE, we mapped cellular diversity, identified six malignant epithelial subclusters and highlighted a GPRC5A^+^ malignant epithelial subpopulation with high developmental potential. Leveraging bulk RNA−seq data from the TCGA−LUAD cohort, we built an 8−gene risk model via LASSO−Cox regression and functionally validated one key component, FAM189A2, as a tumor suppressor *in vitro*. These findings enrich our understanding of lung cancer biology and provide a practical framework for risk stratification and therapeutic targeting.

## 2 Materials and methods

### 2.1 Data collection and preprocessing

scRNA-seq data for lung cancer were obtained from GEO (GSE196303, 13 samples, accessed May 26, 2025;GSE131907,58 samples; accessed Nov 20, 2025).

Count matrices were processed with Scanpy v1.9.1 (10). Doublets were identified using Scrublet and excluded from downstream analyses (11). Bulk RNA-seq data and corresponding clinical annotations for LUAD were retrieved from TCGA (accessed May 23, 2025) and analyzed with limma v3.52 (12).

### 2.2 scRNA-seq clustering, visualization and cell annotation

Cells with <300 or >8,000 detected genes, mitochondrial transcripts >10%, or <1,000 UMIs were filtered out. Library sizes were normalized to 10,000 counts per cell (*sc.pp.normalize_total*, *target_sum=1e4*) and log-transformed. Highly variable genes (n=2,500) were selected (*sc.pp.highly_variable_genes*), followed by PCA (40 components). Batch effects were mitigated using Harmony integration (13). Graph-based clustering was performed with the Leiden algorithm (resolution=0.2) (14), and embeddings were visualized with UMAP. Cluster annotation was guided by literature-curated markers and refined by differential expression using *sc.tl.rank_genes_groups* (Wilcoxon rank-sum).

### 2.3 InferCNV analysis

To infer large-scale copy-number alterations at single-cell resolution, we used inferCNVpy (v0.3.0) on the lung cancer scRNA-seq dataset. Gene-level genomic coordinates were derived from the GRCh38 GTF annotation using the *genomic_position_from_gtf* function, and raw UMI count matrices together with cell-type labels were supplied as inputs. Immune cell populations (T cells, B cells, monocytes, and NK cells) were specified as non-malignant reference categories. Copy-number profiles were smoothed along each chromosome using a sliding window of 101 genes (window_size=101), while other normalization and denoising parameters followed the inferCNVpy defaults.

For each cell, inferCNVpy computed a continuous CNV-burden score (cnv_score) summarizing the magnitude of inferred copy-number deviations relative to the reference cells. Inspection of the CNV-score distribution across the cohort revealed a low-scoring mode corresponding to reference-like cells and a higher-scoring tail. We therefore used a threshold of 0.025. Malignant epithelial cells were then subjected to dimensionality reduction (PCA and UMAP), k-nearest-neighbor graph construction, and Leiden clustering to resolve intratumoral CNV-defined subpopulations.

### 2.4 Gene set enrichment analysis and gene ontology enrichment analysis

Gene set enrichment analysis (GSEA) and GO enrichment were performed with clusterProfiler (15). Ranked gene lists were queried against curated pathway collections; GO analysis was restricted to the Biological Process ontology. Multiple testing was controlled by the Benjamini–Hochberg method, and pathways with adjusted *P* < 0.05 were considered significant.

### 2.5 Subcluster stemness analysis and trajectory analysis

We applied CytoTRACE2 to estimate per-cell differentiation (higher score = less differentiated) across subclusters (16). Scores were summarized per subcluster (median, IQR) and compared using the Kruskal–Wallis test with Benjamini–Hochberg correction for *post hoc* contrasts. Cells in the upper decile of CytoTRACE2 defined the putative root population.

### 2.6 Transcription factor regulon analysis

Transcription factor (TF) regulons in malignant epithelial cells were reconstructed using the three−step pySCENIC workflow (17). In the first step, gene regulatory networks were inferred with the pyscenic grn command using the GRNBoost2 method and a curated list of 1,839 human TFs as candidate regulators. In the second step, co−expression modules were pruned to direct TF–target regulons by motif enrichment analysis with the human hg38 cisTarget ranking databases and the corresponding motif annotation table (motifs−v10nr_clust−nr.hgnc−m0.001−o0.0.tbl), using the pyscenic ctx command with the *–mask_dropouts* option. These databases restrict motif ranking to genomic regions approximately ±10 kb around annotated transcription start sites. We retained only “activating” modules that did not contain “weight>50.0%” flags in the context annotation. For motif enrichment, we required a normalized enrichment score (NES) ≥ 3.0 and either direct gene annotation or orthology to a directly annotated gene, and we discarded regulons containing fewer than 10 target genes. In the third step, regulon activity was quantified at single−cell resolution using AUCell, yielding an AUC matrix for all regulons across all malignant epithelial cells. AUC scores were binarized with AUCell−derived thresholds to define “on/off” regulon states, and both the binarized regulon matrix and AUC scores were imported into the AnnData. Cluster−specific regulons among malignant epithelial subclusters were identified by computing Z−scored regulon activity per cluster relative to the global mean and standard deviation; regulons with Z ≥ 0.2 and high regulon specificity scores (RSS) were considered cluster−enriched. These regulons were visualized using heatmaps of Z−scores and RSS−based rank plots, and TF–target networks were inspected to highlight dominant regulatory programs.

### 2.7 Construction and validation of a novel prognostic risk model

The top 200 genes from the GPRC5A^+^ epithelial cluster were subjected to univariate Cox regression against overall survival in TCGA-LUAD; genes with *P* < 0.05 proceeded to LASSO-Cox modeling. Patient-level risk scores were computed as, Risk score = Σ(Coef_i_ × Expr_i_). Patients were dichotomized at the median risk score into high- and low-risk groups. Prognostic performance was evaluated by Kaplan–Meier analysis with log-rank testing and time-dependent ROC curves.

### 2.8 Immune infiltration analysis and functional enrichment analysis

Bulk expression profiles were transformed to log2(TPM + 1) and deconvolved with CIBERSORT using the LM22 leukocyte signature (1,000 permutations) (18). To evaluate immune evasion potential, TIDE (Tumor Immune Dysfunction and Exclusion) scores were computed for each sample under default parameters.

### 2.9 Drug sensitivity assessment

Predicted drug responses were inferred with pRRophetic (v0.5) trained on the GDSC2_Expr reference set (19). Study expression matrices were converted to log2(TPM + 1), mapped to HGNC symbols, and gene-wise z-scored to match the training distribution. Batch effects were corrected using the package’s built-in ComBat option. For each compound, ridge-regression models estimated individual IC50 (log10 µM) values; predictions were repeated across random seeds and summarized by the median per sample.

### 2.10 Cell line culture, FAM189A2 knockdown/overexpression, and qPCR validation

A549 cells (Wuhan Pricella) were cultured in Ham’s F−12K supplemented with 10% FBS (Gibco) and 1% penicillin/streptomycin at 37 °C in 5% CO_2_ (~95% humidity); NCI−H23 cells were maintained in RPMI−1640 with 10% FBS under identical conditions. Cell identity was confirmed by STR profiling and all lines tested mycoplasma−negative prior to use. For loss−of−function, shRNAs targeting FAM189A2 (sh−1: GCTCGAATCAAAGGTGTGGAA; sh−2: GCCTTGAAACTCTTTCCGGTT) were cloned into pLKO.1−TRC, packaged in HEK293T cells, and viral supernatants collected at 48 h. A549 cells were transduced (8 µg/mL polybrene) and selected with puromycin (2 µg/mL) to establish stable knockdown pools; a non−targeting shRNA served as negative control. Knockdown efficiency was verified by qPCR. For gain−of−function, the 1,824−bp FAM189A2 coding sequence was inserted into pcDNA3.4 to generate pcDNA3.4−FAM189A2; empty vector (EV) was used as a matched control. Transient overexpression was performed at 80% confluence in 6−well plates. Where indicated, stable pools were generated by G418 selection at the pre−determined kill concentration (600 µg/mL for 3 days) and maintained thereafter at half concentration; overexpression was confirmed by qPCR. Total RNA was extracted with TRIzol, reverse−transcribed from 1 µg RNA, and quantified by SYBR−based qPCR on a 96−well platform. Primer sequences (5′ to 3′) were: FAM189A2_F, CGGAATTCGGTCGCCACCATGTACTCCTGGTAAACCTCTTTGTG; FAM189A2_R, CGTCGACTCACAGGACAGTCTCTCGGATGAC; ACTB_F, GAAGTGTGACGTTGACATCCG; ACTB_R, GCCTAGAAGCATTTGCGGTG. Melt−curve analysis confirmed single products. Relative expression was calculated by 2^−ΔΔCt^ using ACTB as the endogenous control. All qPCRs were run in technical triplicates with ≥3 biological replicates. Unless otherwise specified, antibiotics were removed 24 h before functional assays.

### 2.11 Wound healing assay

For scratch assays, confluent monolayers (90%) in 6-well plates were wounded with a sterile 200 µL pipette tip, rinsed twice with PBS, and replenished with fresh medium. Images at 0, 24, and 48 h were analyzed to quantify scratch closure relative to baseline.

### 2.12 Cell invasion assay

Transwell inserts (8 µm) were coated with Matrigel and solidified at 37°C for 6 h, then pre-wetted with 50 µL complete medium for 30 min. After serum starvation (5% FBS medium), 1.5×10^5^ FAM189A2-NC or knockdown cells in 200 µL serum-free medium were added to the upper chamber; the lower chamber contained 600 µL medium with 15% FBS as chemoattractant. After 48 h at 37 °C, non-invading cells were removed. Cells on the underside were fixed and stained with 0.5% crystal violet. Five random fields per insert were imaged at 200× for quantification.

### 2.13 Western blot analysis of EMT markers

Cells were lysed in RIPA buffer with protease/phosphatase inhibitors; protein was quantified by BCA, resolved (20 µg/lane) by SDS–PAGE, and transferred to PVDF membranes. After blocking (5% BSA or 5% non−fat milk, 1 h), membranes were incubated overnight at 4 °C with primary antibodies against E−cadherin (CST <ns/>3195, 1:1000), N−cadherin (CST <ns/>13116, 1:1000), with α/β−Tubulin as loading controls (1:3000–1:5000), followed by HRP−conjugated secondary antibodies (1:5000, 1 h, RT) and ECL detection. Band intensities were quantified in ImageJ, normalized to the loading control, then to the matched control group (shNT for knockdown; EV for overexpression). Data are presented as mean ± SEM from ≥3 biological replicates.

### 2.14 Statistical analysis

Analyses were performed in R v4.2.1. Non-normally distributed variables were compared with the Wilcoxon rank-sum test. Overall survival was assessed using Kaplan–Meier curves with log-rank tests. Two-sided *P* < 0.05 was considered statistically significant.

## 3 Results

### 3.1 Single−cell TME landscapes of lung cancer

**Figure 1:**
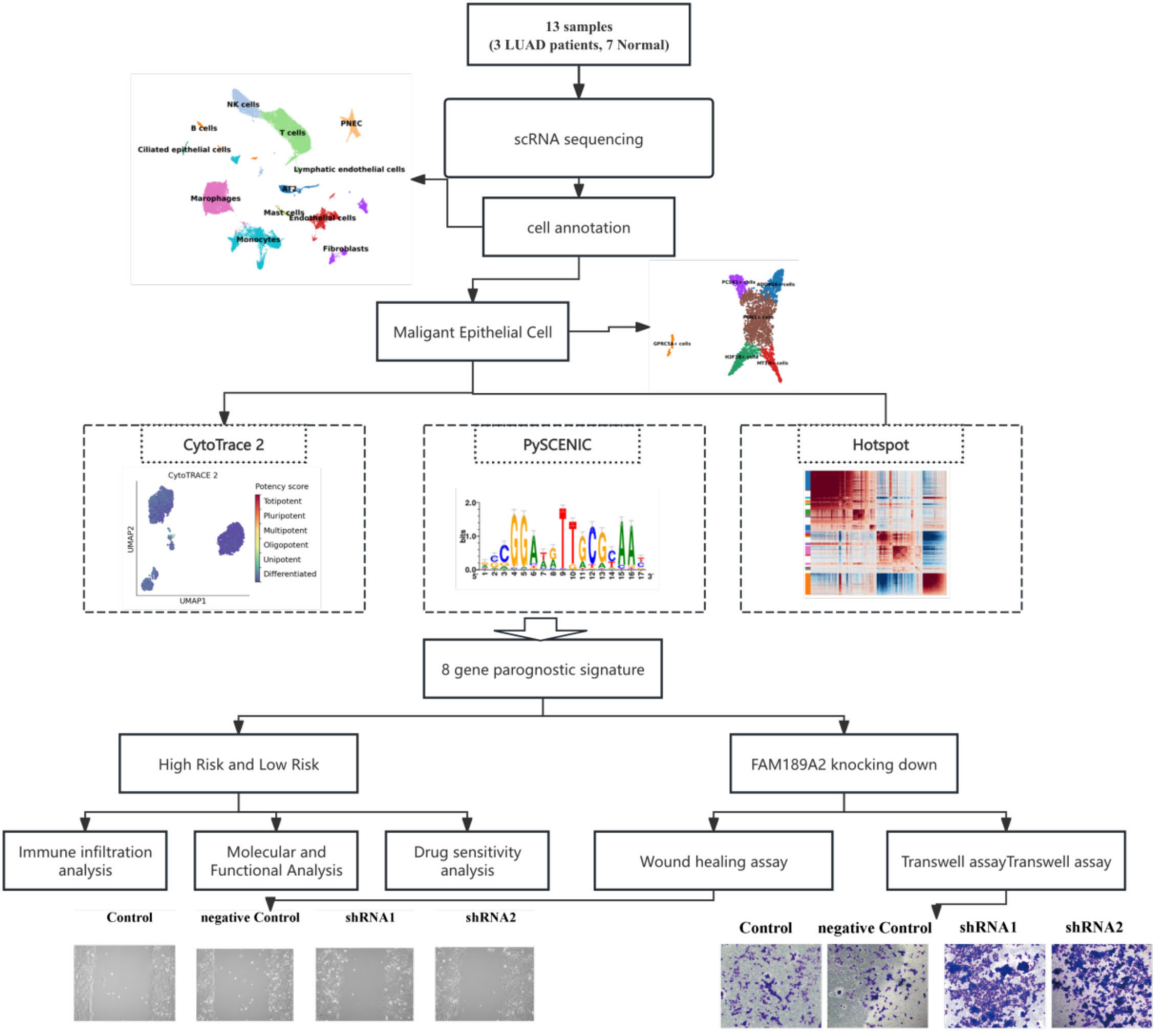
Schematic of overall experimental workflow.

**Figure 2:**
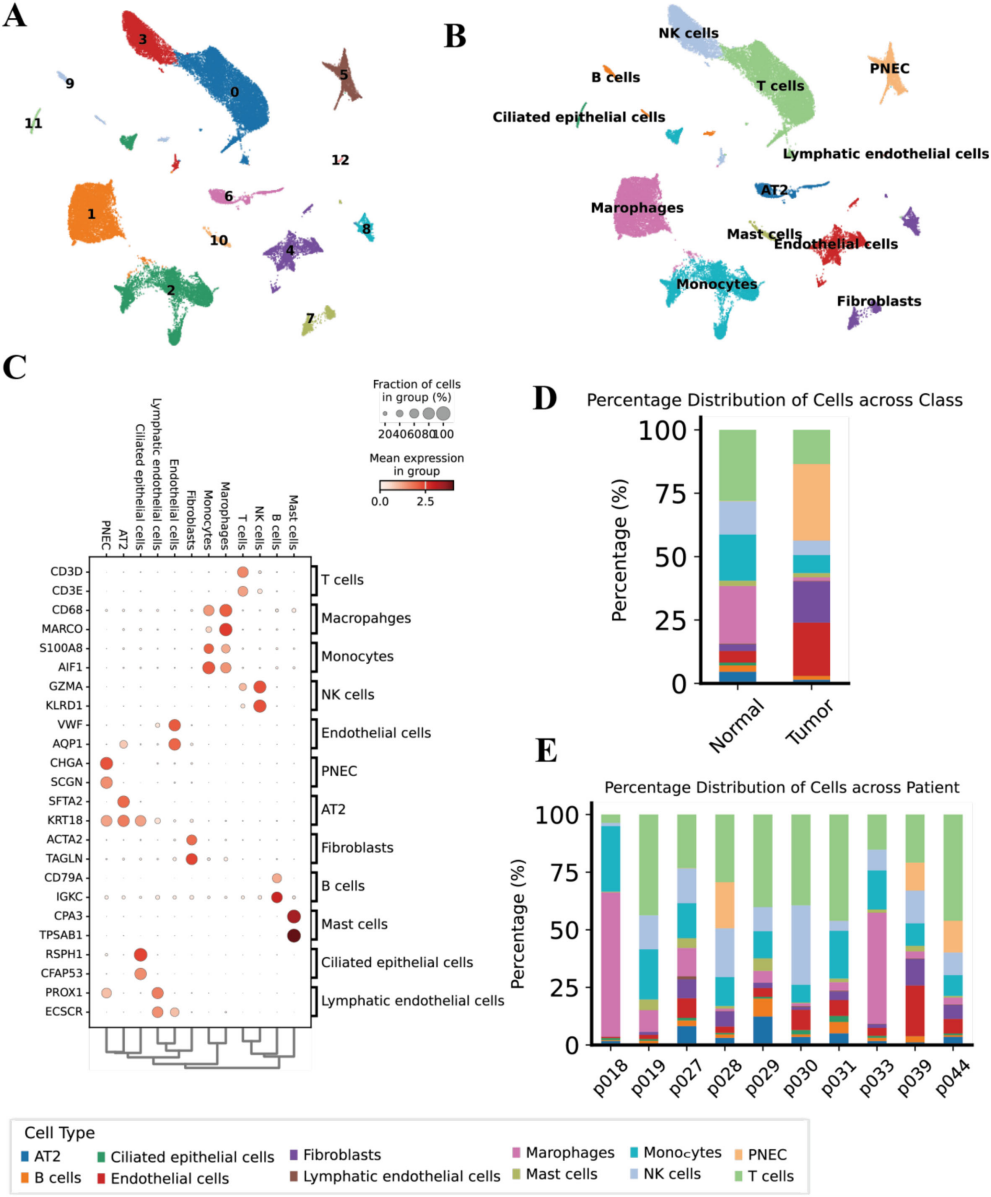
Single-cell landscape of immune and stromal cells in lung cancer **(A)** UMAP of all cells colored by cluster;**(B)** UMAP by annotated cell type. **(C)** Dot plot of canonical marker gene expression. **(D)** Stacked barplot of cell-type composition in tumor vs. normal sample. **(E)** Per-patient cell-type abundances.

We assembled scRNA−seq profiles from 13 lung cancer related samples (10 patients) and, after standard QC and integration, retained 48,149 cells for analysis (**Figure 1**; **Supplementary Table S1**). Unsupervised clustering followed by UMAP revealed 12 transcriptionally distinct clusters that map to three major compartments (**Figures 2A, B**), lymphoid (T, B, NK and plasma cells), myeloid (monocytes, macrophages, mast cells) and stromal/epithelial (vascular and lymphatic endothelial cells, fibroblasts, and lung epithelial cells including AT2, ciliated epithelium and PNEC).

Canonical markers supported these annotations (**Figure 2C**; **Supplementary Table S2**), T cells (CD3D, CD3E), NK cells (NKG7, KLRD1, GZMA), B cells (CD79A, IGKC), macrophages (CD68, MARCO), monocytes (S100A8, AIF1), mast cells (TPSAB1, CPA3), endothelial cells (VWF, AQP1), lymphatic endothelium (PROX1, ECSCR), fibroblasts (ACTA2, TAGLN), alveolar type 2 (AT2)−like epithelial cells (SFTPA2, KRT18), ciliated cells (RSPH1, CFAP53) and PNEC (CHGA, SCGN).

We next compared cellular compositions across tissue sources and individuals. Tumor samples showed a relative expansion of fibroblasts and PNEC, whereas monocytes tended to be more abundant in adjacent normal lung, overall patterns were reproducible across patients (**Figures 2D, E**). This single−cell atlas establishes the cellular baseline of the lung cancer TME and motivates a focused interrogation of the epithelial compartment to distinguish malignant from non−malignant epithelial cells in the next section.

### 3.2 Identification and functional characterization of malignant epithelial cells

**Figure 3:**
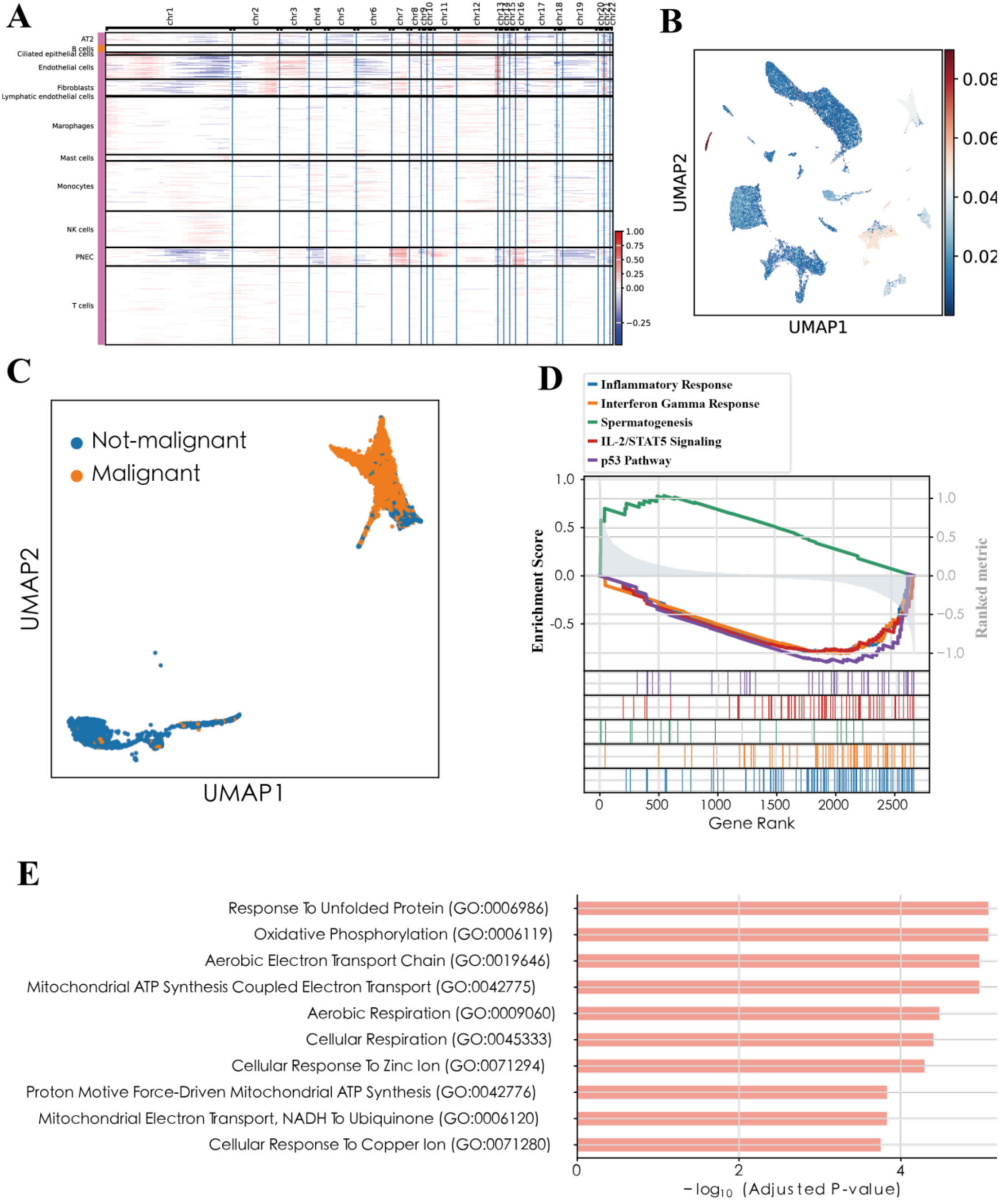
Identification of malignant cell subpopulations in lung cancer **(A)** Heatmap of inferred copy-number variation (CNV) profiles across epithelial cells. **(B)** UMAP colored by CNV score. **(C)** UMAP with malignant vs. non-malignant epithelial cells labeled. **(D)** GSEA comparing malignant vs. normal epithelial cells. **(E)** GO Biological Process terms upregulated in malignant cells.

Using inferCNV on the epithelial compartment, we observed broad, segment−level copy−number shifts in tumor−derived epithelium but not in epithelium from non−tumor sources (**Figure 3A**). A per−cell CNV score projected onto UMAP cleanly separated these two states (**Figure 3B**), enabling a stringent classification of malignant and non−malignant epithelial cells (**Figure 3C**).

Transcriptomically, malignant epithelial cells upregulated proliferation and stress−adaptation programs, whereas non−malignant cells retained lineage and homeostatic functions. Hallmark GSEA (malignant vs. non−malignant) highlighted Inflammatory Response and Interferon−γ Response as preferential in non−malignant epithelium, while malignant cells showed positive enrichment for Spermatogenesis (**Figure 3D**). GO−BP analysis of malignant−up genes emphasized proteostasis and metabolic rewiring (unfolded−protein response, protein catabolic processes, mitochondrial/oxidative programs), whereas non−malignant−up genes mapped to ciliation and surfactant biology (**Figure 3E**).

Consistent with these pathway−level differences, malignant cells expressed higher TOP2A, MKI67, STMN1, and NUPR1, while non−malignant epithelial cells maintained differentiation markers such as SFTPC and FOXJ1. Together, these results define a robust malignant epithelial state marked by CNV burden and proliferative/metabolic programs, setting the stage to ask which malignant transcriptional modules track with patient prognosis in bulk lung cancer cohorts.

### 3.3 Co−expression modules in malignant cells and their prognostic significance

**Figure 4:**
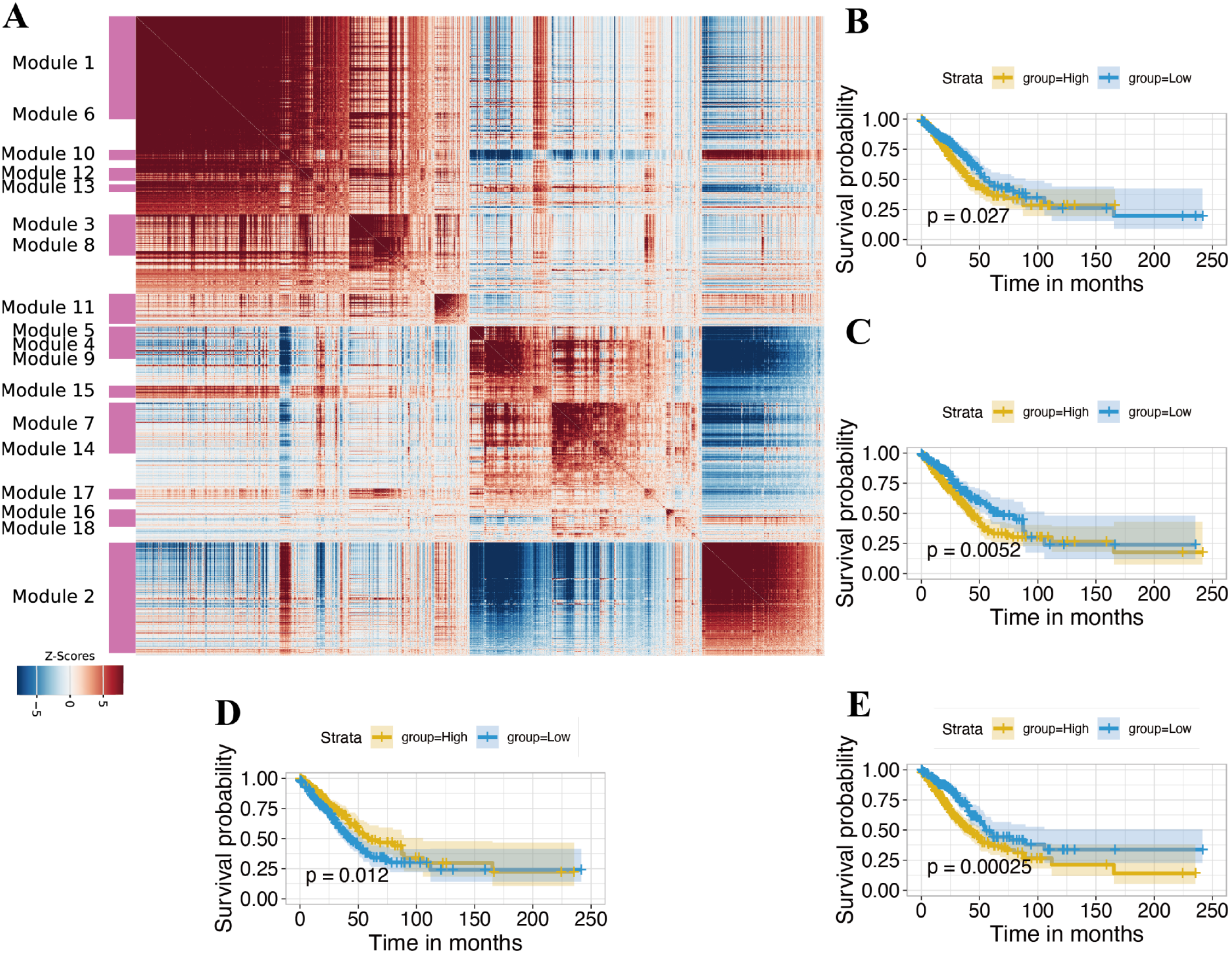
Hotspot analysis **(A)** Hotspot analysis of malignant epithelial cells, showing identified gene co-expression modules in UMAP space. **(B–E)** KM survival curves for TCGA-LUAD patients stratified by high vs. low module scores for module 8, 14, 15, and 16 respectively, demonstrating prognostic impacts.

To resolve malignant cell programs, we applied Hotspot to malignant epithelial cells and identified 18 robust co−expression modules (**Figure 4A**, **Supplementary Table S3**). These modules represent recurrent transcriptional programs across tumor cells. Among them, four modules showed particularly clear biology. Module 16 corresponds to a canonical cell−cycle/DNA−replication program, marked by genes such as CENPK, E2F1, TYMS, TOP2A, NUSAP1 and other mitotic regulators. Module 8 represents an inflammatory and secretory epithelial program enriched for mucins, chemokines and proteases (PIGR, MUC4, MUC5AC, CXCL1/3/5/8, LCN2, MMP7, PLAU), together with extracellular−matrix−remodeling factors. Module 14 is a smaller module containing genes linked to cytoskeletal organization and small secreted peptides (ARPC5L, MYL6B, TMSB15A, CENPH, NPPB). Module 15 captures a signaling and cytoskeletal−remodeling program featuring RTK/IGF1R−related signaling, actin dynamics and vesicle trafficking components, as reflected by genes such as PLOD2, IGF1R, CRK, VASP, SKAP2 and SNX1.

We next projected each module’s gene set into the TCGA−LUAD cohort and computed per−tumor module scores. Kaplan–Meier analyses showed that high scores for the cell−cycle/DNA−replication module (Module 16), for the inflammatory/secretory epithelial module (Module 8), and for the smaller cytoskeletal/secreted−peptide module (Module 14) were each associated with significantly worse overall survival, consistent with their proliferative and tissue−remodelling biology. In contrast, higher activity of Module 15, the RTK/IGF1R−linked signaling and cytoskeletal−remodeling program correlated with improved survival (**Figures 4B–E**).

### 3.4 Heterogeneity of malignant epithelial subpopulations and identification of a key early GPRC5A+ subcluster

**Figure 5:**
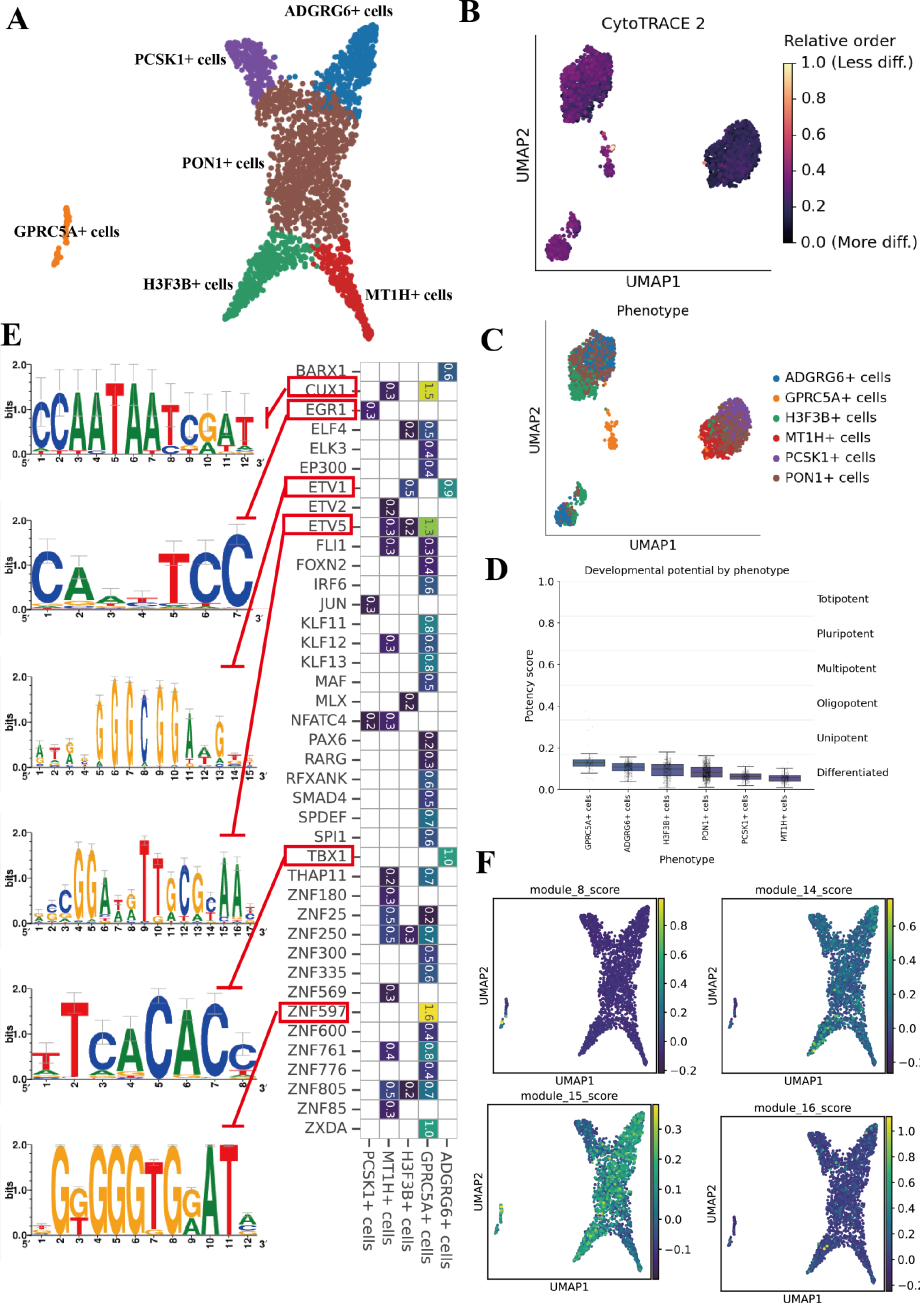
Malignant epithelial subpopulations analysis **(A)** UMAP of malignant epithelial cells, colored by subcluster. **(B)** CytoTRACE scores of each subcluster (higher = more stem-like). **(C)** Cell-cycle phase distribution by subcluster. **(D)** boxplot of top differentially expressed genes for each subcluster. **(E)** Transcription factor regulon activity scores per subcluster. **(F)** Module score profile of each subcluster.

Re−clustering of malignant epithelial cells resolved 6 robust subpopulations, labelled by top markers as ADGRG6^+^, GPRC5A^+^, H3F3B^+^, MT1H^+^, PCSK1^+^ and PON1^+^ (**Figure 5A**). These phenotypes likely represent distinct differentiation lineages or metabolic adaptations within the malignant epithelial compartment. To compare developmental potential, we first computed CytoTRACE scores. The GPRC5A^+^ subpopulation showed the highest CytoTRACE values (least differentiated), whereas PON1^+^ and MT1H^+^ cells scored lower, consistent with a more differentiated state (**Figures 5B–D**). To further place these states along a continuum, we applied diffusion−pseudotime (DPT) analysis. The GPRC5A^+^ cluster occupied the earliest pseudotime positions with the lowest dpt_pseudotime values, from which more proliferative or stress−adapted clusters appeared to emerge (**Supplementary Figure S1A**). Together, CytoTRACE and DPT support the view that GPRC5A^+^ cells represent an early, primed malignant state with high developmental potential rather than a terminally differentiated endpoint.

Regulatory network inference by pySCENIC further distinguished the six phenotypes, recovering transcription−factor regulons with subtype−specific activity, including CUX1, EGR1, ETV2, TBX1, ZNF507 and others (motif logos and regulon AUC heatmap in **Figure 5E**). These regulons offer hypotheses for upstream drivers of the alveolar−like GPRC5A^+^ state versus the more proliferative or hypoxic programs in other clusters. We next asked how the prognostic Hotspot modules map onto these subpopulations. UMAP overlays revealed that Module 15—the RTK/IGF1R−linked signaling and cytoskeletal−remodeling program—is maximally enriched within the GPRC5A^+^ region, whereas Modules 8, 14 and 16, which capture inflammatory/secretory and cell−cycle programs, preferentially mark other malignant subpopulations and show little overlap with GPRC5A^+^ cells (**Figure 5F**). This spatial pattern links the GPRC5A^+^ early malignant state to the favorable−prognosis Module−15 program and, conversely, associates Modules 8/14/16 with more aggressive malignant states.

To assess whether a similar architecture exists in lung adenocarcinoma, we analyzed an independent LUAD scRNA−seq dataset (GSE131907). After quality control, clustering and identification of malignant epithelial cells, we projected the Module−15 gene set onto this dataset. Module−15 scores showed clear spatial heterogeneity on the LUAD UMAP (**Supplementary Figure S1B**) and were highest in malignant cluster 11 (**Supplementary Figure S1C**). This cluster also exhibited high similarity to our discovery−cohort GPRC5A^+^ signature, indicating an analogous GPRC5A−high population in LUAD. Thus, the coupling between an early GPRC5A−high malignant state and the Module−15 signaling program is reproducible across independent single−cell datasets, supporting the relevance of these carcinoid−derived malignant programs to LUAD biology.

Together, these analyses define a continuum of malignant epithelial states in lung cancer and LUAD, from proliferative and stress−adapted subpopulations dominated by Modules 8, 14 and 16 to an early GPRC5A^+^, stem−leaning subpopulation that preferentially engages the Module−15 signaling program.

### 3.5 Prognostic risk model derived from the GPRC5A+ tumor subcluster

**Figure 6:**
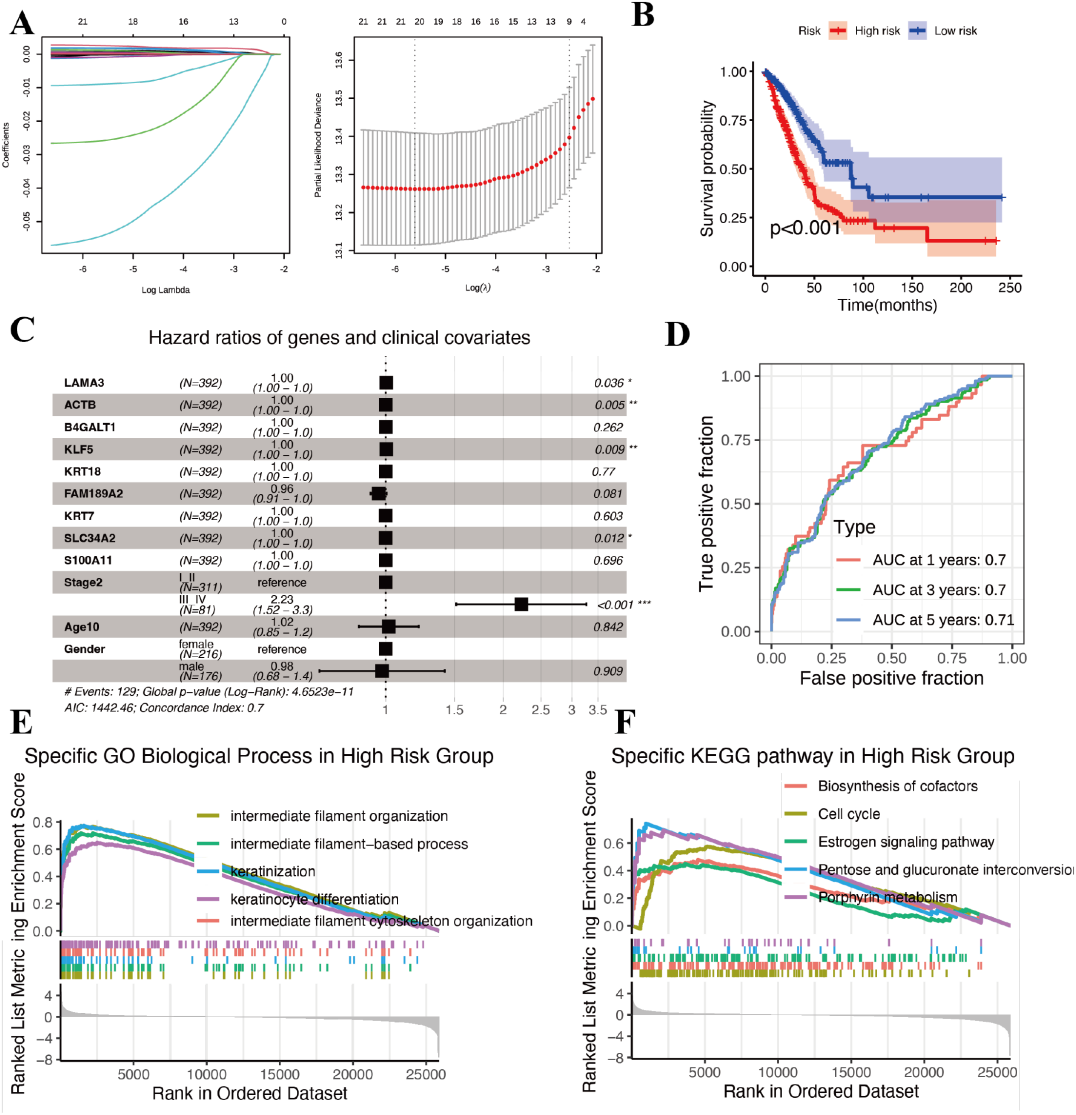
Construction and evaluation of the prognostic risk model. **(A)** LASSO coefficient profiles of candidate genes (left) and ten-fold cross-validation error plot (right). **(B)** KM overall survival curves for TCGA-LUNG CANCER patients stratified by the 8-gene risk score. **(C)** Multivariable Cox regression forest plot of the 8 genes in the signature. **(D)** Time-dependent ROC curves for 1, 3, and 5-year OS prediction by the 8-gene risk score. **(E)** GSEA of GO Biological Process terms. **(F)** GSEA of KEGG pathways highlighting differences.

Guided by the GPRC5A+ subcluster, we used its top genes to build a transcriptomic risk model in TCGA−LUAD. LASSO−Cox identified 8 genes with non−zero coefficients, LAMA5, ACTB, B4GALT1, KLF5, KRT18, FAM189A2, SLC34A2, S100A11, using 10−fold cross−validation for λ selection (**Figure 6A**). A risk score was computed per patient as Σ(β_i_ × expression_i_), and the cohort was median−split into High− and Low−risk groups. This signature robustly stratified survival: the High−risk group had significantly worse OS than the Low−risk group (*P* < 0.001; **Figure 6B**). Time−dependent ROC analysis yielded AUC ≈ 0.70 at 1, 3, and 5 years, indicating stable performance across follow−up windows (**Figure 6D**). To further assess the robustness and generalizability of this 8−gene signature, we applied it to two independent NSCLC/LUAD microarray cohorts: GSE30219 (NSCLC, n = 293 tumors) and GSE31210 (stage I–II LUAD, n = 226 patients). In each cohort, patients were stratified into high− and low−risk groups according to the 8−gene risk score, and the low−risk group consistently showed significantly better OS than the high−risk group (**Supplementary Figures S2A, B**). These external validations support the prognostic value of the 8−gene signature beyond the TCGA training set.

To dissect the relative contributions of individual genes and clinical variables, we fitted a multivariable Cox model including the 8 signature genes together with age, gender and stage (**Figure 6C**). In this model, most genes had modest effect sizes with hazard ratios close to 1.0 per unit increase in expression, whereas advanced stage (III–IV vs. I–II) showed the expected strong risk effect. FAM189A2 displayed a borderline protective tendency (HR ≈ 0.96, 95% CI 0.91–1.00, P = 0.081), which is directionally consistent with its enrichment in the favorable Module−15 RTK/IGF1R−linked signaling program within the GPRC5A^+^ subcluster.

Collectively, the 8−gene signature translates our single−cell findings into a clinically interpretable tool that separates lung cancer into prognostically distinct groups.

### 3.6 Transcriptomic, immune, and drug−sensitivity differences between high− and low−risk groups

**Figure 7:**
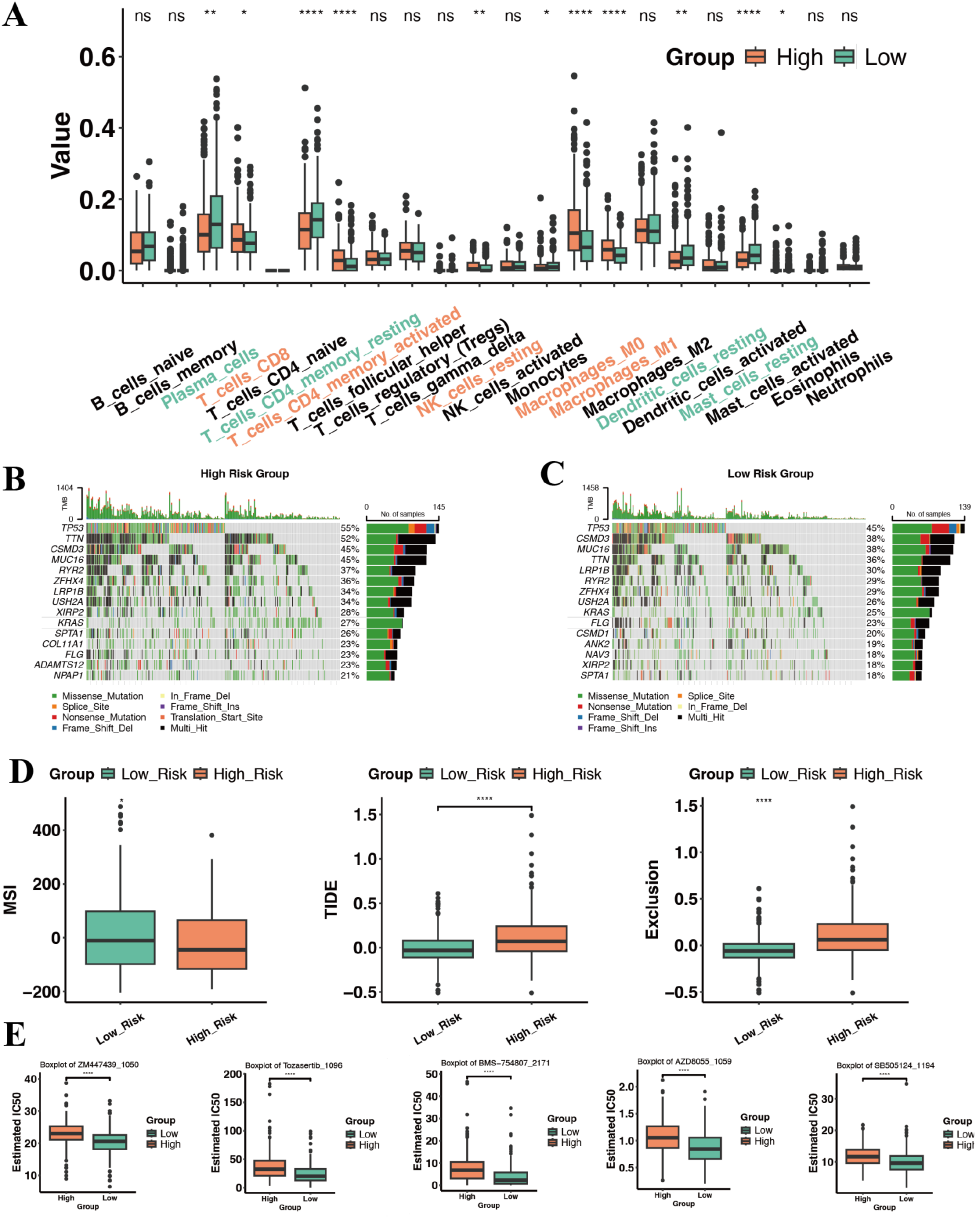
Functional differences between high- and low-risk groups. **(A)** Boxplot of CIBERSORT-estimated abundances of 22 immune cell types. **(B, C)** Oncoprint-style mutation plots for High-risk **(B)** and Low-risk **(C)** groups, showing the top mutated genes in each. **(D)** Boxplots of MSI scores and TIDE scores in High vs. Low groups. **(E)** Boxplots of predicted drug sensitivities (IC50) for selected compounds. Significance levels were denoted as *P < 0.05, **P < 0.01, ****P < 0.0001.

Having stratified TCGA−LUAD patients with the 8−gene signature, we compared the two risk groups across transcriptional programs, tumor immunity, somatic alterations and predicted drug response. Pathway comparison further clarified their biology. High−risk tumors were enriched for keratinisation, keratinocyte differentiation and intermediate filament cytoskeleton (**Figure 6E**), and for estrogen signaling, pentose and glucuronate interconversions and porphyrin metabolism (**Figure 6F**). These results suggest a shift toward epithelial remodeling and specific metabolic reprogramming in high−risk disease, whereas low−risk tumors retain more alveolar−differentiation−like features. Deconvolution of bulk expression revealed marked differences in immune composition between risk groups. High−risk tumors showed higher fractions of CD8^+^ T cells, activated memory CD4^+^ T cells, macrophages (M0/M1) and resting NK cells, whereas low−risk tumors were enriched for plasma cells, resting memory CD4^+^ T cells, resting dendritic cells and resting mast cells (**Figure 7A**). At first glance, this immune−inflamed phenotype appears at odds with the poorer survival of the high−risk group. Side−by−side oncoplots showed broadly similar driver mutation landscapes across groups (**Figures 7B, C**). Microsatellite instability (MSI) tended to be higher in low−risk tumors (**Figure 7D**). High−risk tumors also exhibited higher TIDE and Exclusion scores, indicating greater potential for T−cell exclusion.

To further reconcile the apparent discrepancy between an immune−rich high−risk group and its poor survival, we quantified T−cell exhaustion and stromal signatures in TCGA−LUAD. At the single−gene level, several checkpoint/exhaustion markers (PDCD1, LAG3, CTLA4, TIGIT, HAVCR2, PDCD1LG2 and CD274) showed only modest, directionally variable differences between high− and low−risk tumors (**Supplementary Figure S3A**). Consistently, an exhaustion−related ssGSEA signature was not significantly different between risk groups (P = 0.11), and a myeloid/bone−marrow ssGSEA score also showed no clear separation (P = 0.22; **Supplementary Figure S3B**). In contrast, a matrix/cancer−associated fibroblast (CAF)−related ssGSEA signature was significantly enriched in high−risk tumors (P = 2.8 × 10^-4^; **Supplementary Figure S3B**), indicating a more pronounced stromal/ECM activation in this group. Taken together with the higher TIDE Exclusion scores, these findings suggest that high−risk tumors are immune−enriched but embedded in a matrix−rich, CAF−dominated microenvironment, compatible with an “inflamed yet restrained” TME rather than a uniformly favorable immune−hot state.

Using expression−based pharmacogenomic modelling, low−risk tumors showed lower estimated IC_50_ values for several agents targeting cell−cycle and growth−factor pathways, ZM447439 and Tozasertib (Aurora kinase inhibitors), BMS−754807 (IGF−1R/IR inhibitor), AZD8055 (mTOR inhibitor) and SB505124 (TGF−βRI inhibitor), indicating higher predicted sensitivity (**Figure 7E**). High−risk tumors were relatively resistant to these agents, in line with their proliferative and stromally remodeled phenotype. Overall, these orthogonal readouts help explain the survival difference between risk groups and point to distinct therapeutic strategies: low−risk LUADs may benefit more from targeting Aurora kinase, IGF−1R, mTOR and TGF−β pathways, whereas high−risk tumors are characterized by greater immune restraint within a dense matrix and may require combination strategies that simultaneously modulate the stroma and reinvigorate anti−tumor immunity.

### 3.7 Identification of FAM189A2 as a hub gene in the risk signature

**Figure 8:**
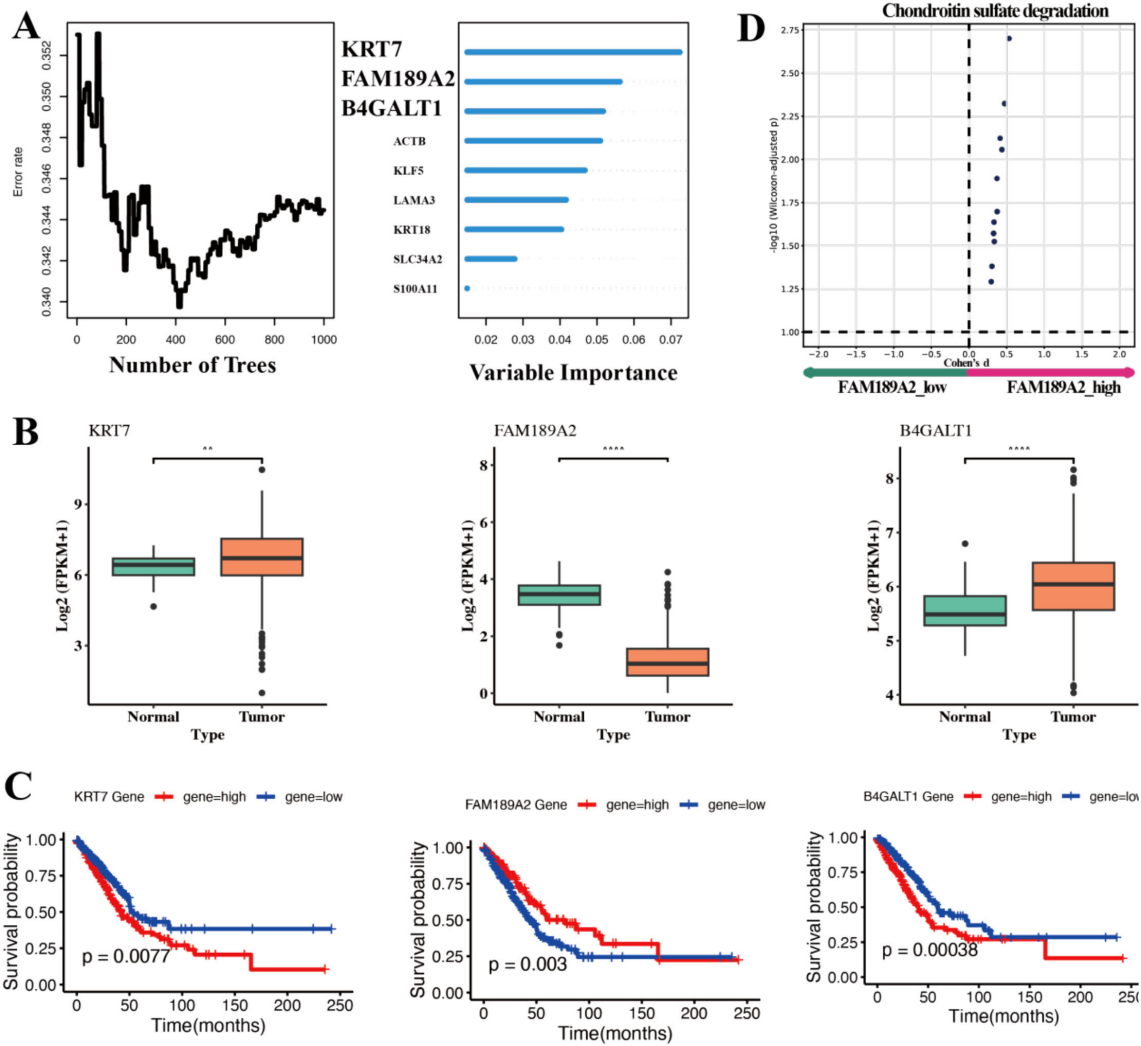
Identification of FAM189A2 as a key hub gene. **(A)** Variable importance scores from Random Forest analysis of candidate genes. **(B)** Boxplots comparing expression of ACTB, FAM189A2, B4GALT1 in normal lung vs. LUAD tumor tissues. **(C)** KM overall survival curves for patient subsets with high vs. low expression of the three genes. Significance levels were denoted as **P < 0.01, ****P < 0.0001.

To pinpoint potential drivers within the signature, we trained a Random Forest classifier on the 8 signature genes together with closely co−expressed epithelial markers. The model reached a stable out−of−bag error with 1,000 trees (**Figure 8A**) and consistently ranked KRT7, FAM189A2 and B4GALT1 as the top predictors. Expression analyses using TCGA confirmed tumor downregulation of FAM189A2 and tumor−upregulation of KRT7 and B4GALT1 (**Figure 8B**). In an independent GEO dataset (GSE17558), FAM189A2 expression was likewise higher in adjacent non−tumor tissue than in matched tumor samples (**Supplementary Figure S2C**), providing external support for its tumor−suppressive pattern. Kaplan–Meier curves further showed that high FAM189A2 expression predicts better survival (P = 0.003), whereas high KRT7 and high B4GALT1 expression predict worse survival (P = 0.0077 and P = 0.00038, respectively; **Figure 8C**). Together, the machine−learning importance ranking, tumor–normal contrasts across TCGA and GSE17558, and survival analyses converge on FAM189A2 as a protective hub within the risk framework, in line with its enrichment in the GPRC5A^+^ malignant subpopulation.

### 3.8 Functional characterization of FAM189A2 in lung cancer cell migration and invasion

**Figure 9:**
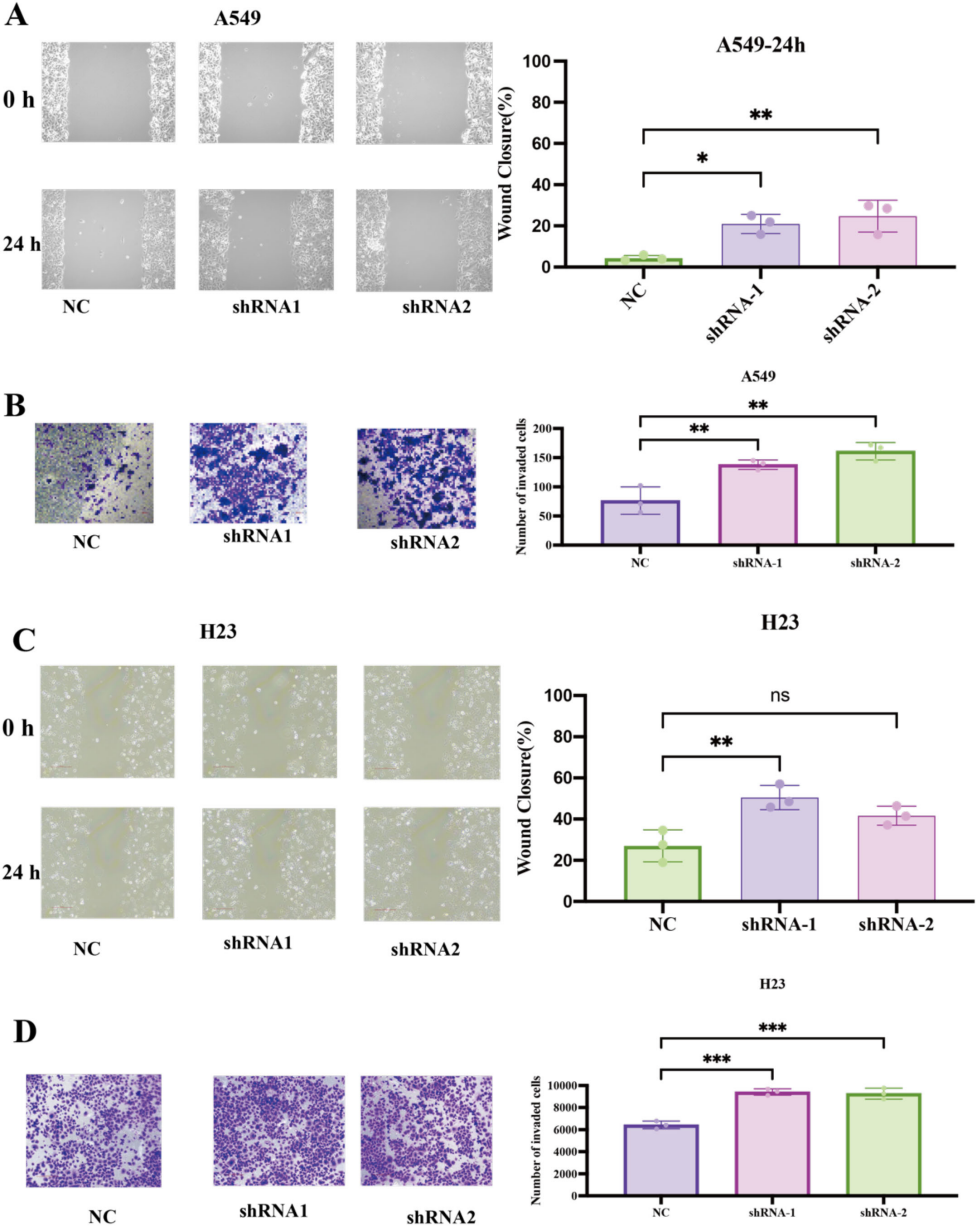
Effects of FAM189A2 knockdown on lung cancer cell migration and invasion. **(A, B)** A549 cells transduced with non−targeting control (shNC) and two independent FAM189A2 shRNAs (sh−1, sh−2). **(A)** Wound−healing assay with representative images at 0 and 24 h **(B)** Matrigel Transwell invasion assay with representative images of crystal−violet−stained invaded cells and quantification of invaded cells per field. **(C, D)** NCI−H23 cells with the same shRNA constructs. **(C)** Wound−healing assay. **(D)** Matrigel invasion assay. *P < 0.05; **P < 0.01; ***P < 0.001; ns, not significant (*P ≥ 0.05); two−sided tests.

We first tested whether FAM189A2 constrains motility and invasiveness in LUAD cells by knocking it down in two cell lines using independent shRNAs. Efficient knockdown was confirmed by qPCR (**Supplementary Figure S3C**). In A549 cells, FAM189A2−silenced cells closed scratch wounds markedly faster than non−targeting controls at 24 h, indicating enhanced migratory capacity (**Figure 9A**). Consistently, knockdown significantly increased the number of invading A549 cells in Matrigel Transwell assays (**Figure 9B**). The corresponding experiments in NCI−H23 yielded similar results: FAM189A2 knockdown accelerated wound closure and increased invasion compared with shRNA controls (**Figures 9C, D**). In all assays, non−targeting shRNA and parental cells were indistinguishable, suggesting that the phenotype is specific to FAM189A2 depletion.

To examine whether the opposite perturbation produces reciprocal effects, we next overexpressed FAM189A2 in both A549 and H23 cells using a pcDNA3.4−FAM189A2 construct with matched empty−vector controls. Overexpression was verified by qPCR (**Supplementary Figure S3C**). In A549, FAM189A2 overexpression slowed scratch−wound closure and reduced invasion through Matrigel relative to empty−vector cells (**Supplementary Figures S4A, B**). In H23, which is more aggressive at baseline, FAM189A2 overexpression likewise decreased wound closure and significantly reduced the number of invading cells (**Supplementary Figures S4C, D**). Thus, across two LUAD lines and two perturbation directions, the pattern was consistent: lower FAM189A2 was associated with faster migration and greater invasion, whereas higher FAM189A2 dampened these traits.

**Figure 10:**
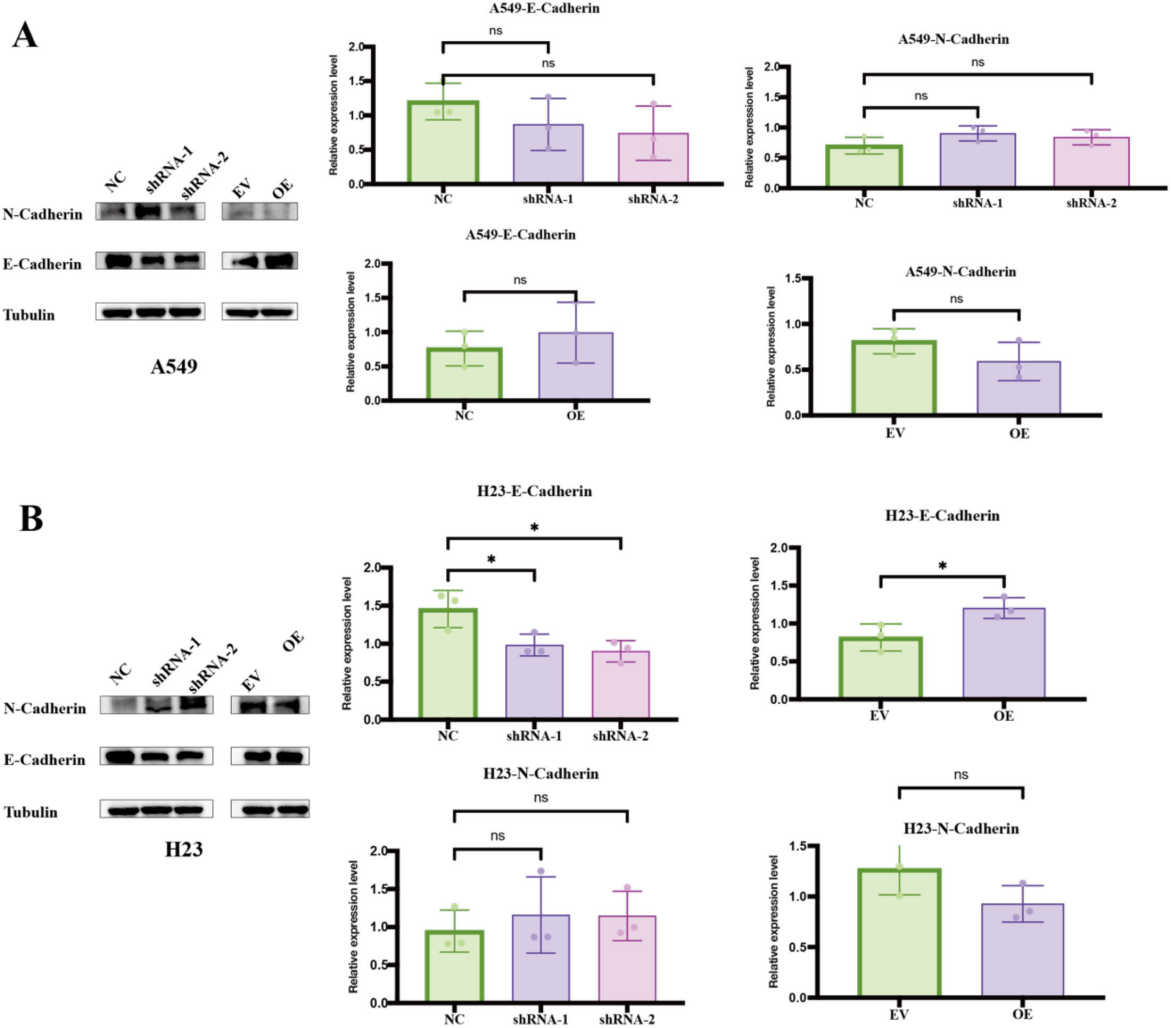
Effects of FAM189A2 knockdown and overexpression on E−cadherin and N−cadherin expression. **(A)** A549 and **(B)** NCI−H23 cells. For each panel, the left subpanel shows Western blots of N−cadherin, E−cadherin and Tubulin in NC, two FAM189A2 shRNAs, EV and FAM189A2−OE cells. The right subpanels show densitometric quantification of E−cadherin and N−cadherin, normalized to Tubulin and then to NC or EV. Significance levels were denoted as *P < 0.05, ns indicates no significant difference.

We further investigated EMT markers by Western blot (**Figures 10A, B**). In A549 knockdown cells, E−cadherin tended to decrease and N−cadherin to increase relative to non−targeting controls, but these changes did not reach statistical significance in our small−n setting and are therefore interpreted as trends (**Figure 10A**). In H23, FAM189A2 overexpression significantly increased E−cadherin protein levels, whereas N−cadherin showed a modest downward trend without achieving formal significance (**Figure 10B**). These EMT−marker patterns, together with the robust effects on migration and invasion, are compatible with a partial EMT shift upon FAM189A2 loss and a converse shift toward a more epithelial phenotype upon its gain, but we regard them as supportive rather than definitive mechanistic proof.

Collectively, bidirectional perturbation of FAM189A2 in two LUAD cell lines demonstrates that reduced FAM189A2 activity is associated with enhanced migratory and invasive behavior, whereas restoring or increasing FAM189A2 dampens these traits. These functional data support the view that FAM189A2 acts as a negative regulator of metastatic potential in lung cancer cells and are directionally consistent with its association with the favorable early GPRC5A^+^ malignant program in our single−cell analyses.

## 4 Discussion

In this study, we integrated single−cell and bulk transcriptomic data to dissect malignant epithelial states in lung cancer and link them to prognosis and potential therapeutic vulnerabilities. At single−cell resolution we identified at least six malignant epithelial subpopulations and several recurrent co−expression modules, ranging from highly proliferative and inflammatory/secretory programs (Modules 8, 14 and 16) to an early GPRC5A^+^ state with high developmental potential that preferentially engages a signaling and cytoskeletal−remodeling program (Module 15). The relative prevalence of these malignant states was then translated into an eight−gene expression signature derived from the GPRC5A^+^ program that stratifies patients into high− and low−risk groups with distinct tumor microenvironments and predicted drug sensitivities.

Our single−cell analysis confirms that intratumoral heterogeneity in lung cancer extends beyond genomic alterations to the level of cell−state diversity (20). Prior studies have shown that lung tumors harbor epithelial cells spanning a continuum from normal−like to fully transformed states. Kim and colleagues profiled lung cancers from early to advanced stages and observed the emergence of undifferentiated cell clusters associated with metastasis (21). Han et al. recently described KRT8^+^ alveolar intermediate cells as a transitional state between normal cells and malignant cells, with stem−like and immune−evasive features and adverse prognosis (22). In our advanced tumors we likewise detected a GPRC5A^+^ subpopulation with high CytoTRACE and early pseudotime, consistent with high developmental potential. However, tumors with stronger engagement of the GPRC5A^+^/Module−15 program showed better outcome, whereas enrichment of proliferative and inflammatory/secretory program (Modules 8, 14 and 16) portended poor survival. One plausible explanation is that, in established tumors, the presence of an early malignant state may indicate that a substantial fraction of cells has not yet fully transitioned into highly aggressive phenotypes, whereas tumors dominated by plastic, KRT8−high transitional or proliferative states are more lethal (22, 23). These nuances underscore that the clinical impact of a given cell state depends on its prevalence and context within a tumor. Our work reinforces the notion that lung cancer is not a single disease but a collection of phenotypically diverse cell populations whose proportions vary between tumors, driving divergent clinical trajectories and treatment responses (24). From a therapeutic standpoint, selectively targeting the most aggressive subpopulations (with strong Module−8/14/16 activity) while sparing or even stabilizing less aggressive states could be beneficial. Single−cell approaches, as used here, are essential for mapping such sublineages and designing state−targeted interventions (25).

An important conceptual question is whether malignant program derived from pulmonary carcinoid tumors are relevant to LUAD. Our discovery scRNA−seq dataset (GSE196303) comprises neuroendocrine tumors and matched normal lung from LUAD patients, whereas our prognostic modelling was performed in LUAD cohorts. We therefore explicitly tested for recurrence of the key malignant program in a histology−matched LUAD single−cell dataset (GSE131907). In that cohort we identified a GPRC5A−high malignant cluster in which the Module−15 signaling program was again preferentially active, mirroring the pattern seen in the discovery dataset. In addition, the eight−gene signature derived from the GPRC5A^+^ program stratified survival not only in TCGA−LUAD but also in two independent NSCLC/LUAD cohorts (GSE30219 and GSE31210). Taken together, these observations suggest that the GPRC5A^+^/Module−15 axis captures a malignant state that is shared across lung tumor histologies, although we acknowledge that cross−histology projection may still introduce bias and therefore interpret these links with appropriate caution.

By combining single−cell insights with bulk deconvolution, we also showed that the high− and low−risk groups differ markedly in immune contexture. High−risk tumors displayed higher fractions of CD8^+^ T cells, activated CD4^+^ memory T cells, NK cells and macrophages, whereas low−risk tumors contained fewer effector lymphocytes and more resting immune populations. At face value this immune−enriched profile seems inconsistent with the poorer survival of the high−risk group. Our additional analyses help reconcile this. Checkpoint and exhaustion markers (PDCD1, LAG3, HAVCR2 and others) and an exhaustion ssGSEA score were not strongly different between risk groups, but a matrix/CAF−related ssGSEA signature and TIDE Exclusion scores were significantly higher in high−risk tumors, indicating dense stromal remodeling and potential physical or biochemical barriers to T−cell function. Thus, the high−risk group appears “inflamed yet restrained”, lymphocytes are present but embedded in a matrix−rich, CAF−dominated milieu, which may blunt their anti−tumor effects (26). This is consistent with pan−cancer analyses showing that immune−rich but stroma−dominated TMEs can fare worse than truly inflamed, T−cell–effective tumors (27). Conversely, the low−risk group is relatively immune−quiescent but less stromally remodeled. These patterns highlight that immune quantity and immune quality are distinct, and that integrating tumor−intrinsic and microenvironmental features may yield more informative biomarkers than PD−L1 expression alone (21, 27, 28). In this context, our eight−gene signature—or the underlying malignant states from which it was derived—could potentially help stratify patients both by prognosis and by likelihood of benefiting from immunotherapy, although this will require prospective validation.

In addition, pharmacogenomic modelling suggested that low−risk tumors are more sensitive to Aurora kinase, IGF−1R, mTOR and TGF−β pathway inhibition, whereas high−risk tumors are relatively resistant. To begin exploring whether these predictions might be reflected at the cell−line level, we performed dose–response assays for three of the predicted agents—Tozasertib (Aurora kinase inhibitor), BMS−754807 (IGF−1R/IR inhibitor) and AZD8055 (mTOR inhibitor), in A549 and NCI−H23 cells. Across a range of concentrations, H23 cells exhibited lower concentration values and greater growth inhibition than A549 for all three drugs (**Supplementary Table S4**), indicating that certain LUAD contexts are intrinsically more susceptible to simultaneous targeting of Aurora, IGF−1R and mTOR signaling. While these *in vitro* experiments are limited to two cell lines and do not directly map onto the high− versus low−risk patient groups, they provide preliminary experimental support for the pharmacogenomic predictions and motivate further testing of pathway−targeted combinations in molecularly defined LUAD subsets. Together with the stromal and immune differences between risk groups, these data may guide hypothesis−generating combination strategies, for example, pairing stroma−modulating agents with immunotherapy in high−risk disease and pathway−targeted agents in tumors with a low−risk−like transcriptional profile.

A particularly novel aspect of our study is the focus on FAM189A2. This gene emerged from our single−cell−derived signature and Random Forest analysis as a candidate protective hub: it is down−regulated in tumors compared with adjacent lung, higher expression is associated with better survival, and this pattern is reproduced in an independent dataset. *In vitro*, bidirectional perturbation in two LUAD cell lines showed that FAM189A2 knockdown consistently enhanced, whereas overexpression dampened, migration and invasion. Changes in EMT markers (decreased E−cadherin and increased N−cadherin upon knockdown, with the converse trend upon overexpression) were modest but directionally compatible with a partial EMT shift. Taken together, these data support a model in which FAM189A2 restrains motility and invasive potential, possibly by stabilizing an epithelial state, although the precise downstream pathways remain to be defined. We therefore regard FAM189A2 as a promising biomarker and putative tumor suppressor that warrants deeper mechanistic and *in vivo* investigation rather than a fully validated therapeutic target at this stage.

This study has several limitations. First, the discovery scRNA−seq cohort (GSE196303) comprises pulmonary neuroendocrine (carcinoid) tumors and matched normal lung from LUAD patients, whereas our prognostic modelling was performed in LUAD, although we observed a similar GPRC5A−high/Module−15 malignant state in an independent LUAD single−cell dataset and validated the eight−gene signature in two external NSCLC/LUAD cohorts, cross−histology projection may still introduce bias. Second, the eight−gene risk model and pharmacogenomic predictions were derived from retrospective cohorts and, despite multivariable adjustment and external validation, overfitting or cohort−specific effects cannot be excluded. Finally, functional data for FAM189A2 are limited to *in vitro* perturbation in two LUAD cell lines, *in vivo* models and deeper mechanistic studies will be required to fully establish its role in lung cancer progression.

By integrating single−cell and bulk transcriptomes, we delineate malignant epithelial programs in lung cancer, identify an early GPRC5A^+^ malignant state linked to a favorable Module−15 signaling program, and derive an interpretable eight−gene signature that stratifies risk in LUAD across multiple cohorts. We further nominate FAM189A2 as a putative tumor suppressor whose reduced expression and activity are associated with a more migratory and invasive phenotype *in vitro*. These results provide a reusable analytic framework and generate testable hypotheses about immune contexture, stromal restraint and pathway−specific vulnerabilities. Prospective validation in histology−matched, independent cohorts, together with spatial profiling and mechanistic studies, will be essential to confirm clinical utility and to refine FAM189A2 and the GPRC5A^+^/Module−15 axis as biomarkers and potential therapeutic entry points.

## Data Availability

The datasets analyzed in this study are publicly available in the NCBI Gene Expression Omnibus (GEO) database under accession numbers GSE131907 (bulk RNA-seq) and GSE196303 (single-cell RNA-seq). Related metadata are provided in the **Supplementary Material**.
