# Supplementary figures and images for: Integration of single cell and bulk transcriptomic analyses identifies FAM189A2 as a key prognostic gene in lung cancer
Source: Front Immunol. 2026 Jan 6;16:1701806. doi: 10.3389/fimmu.2025.1701806 (PMC12816179; doi:10.3389/fimmu.2025.1701806)

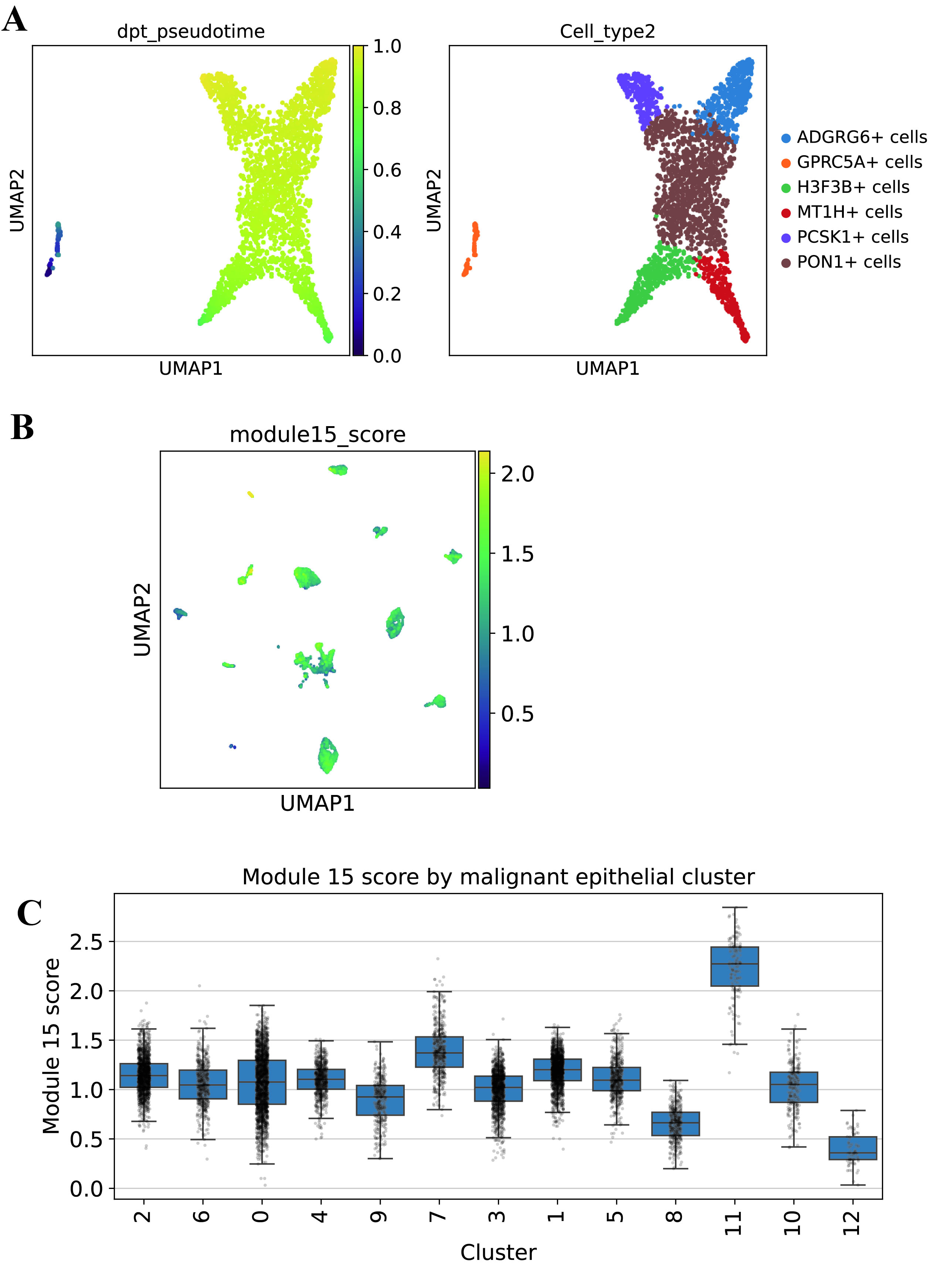

Supplement: Supplementary file 1 [file Image1.jpeg]

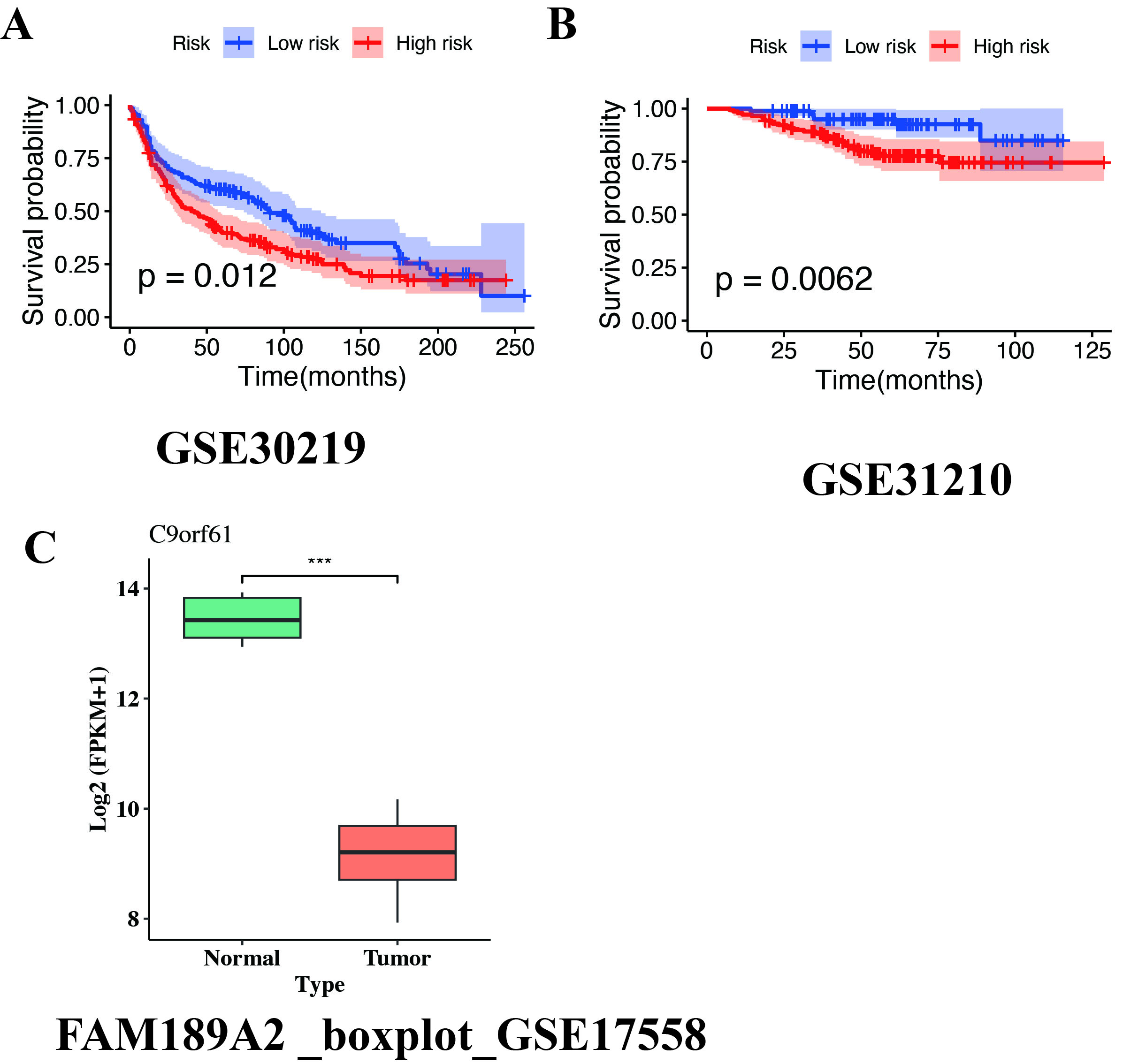

Supplement: Supplementary file 2 [file Image2.jpeg]

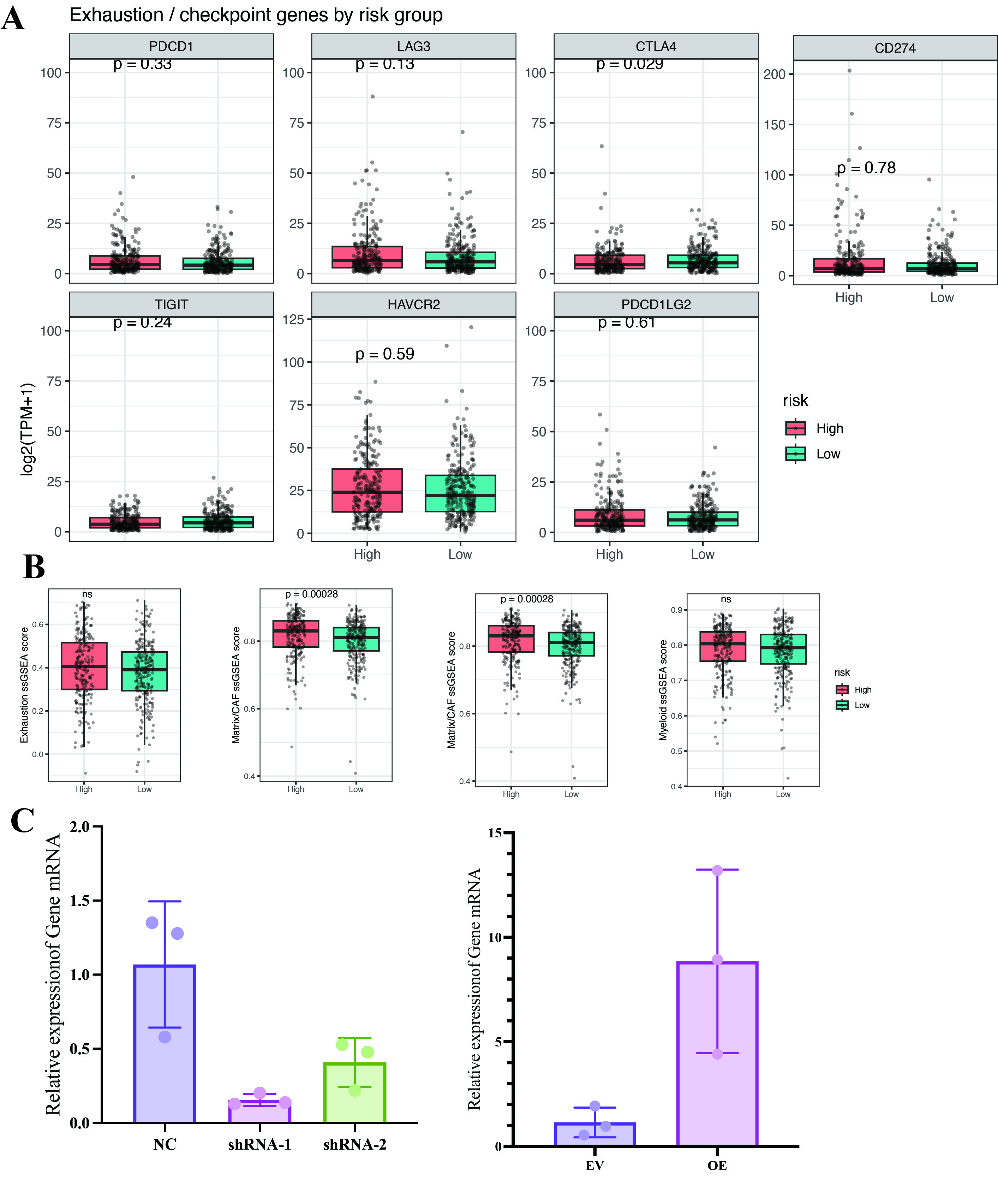

Supplement: Supplementary file 3 [file Image3.jpeg]

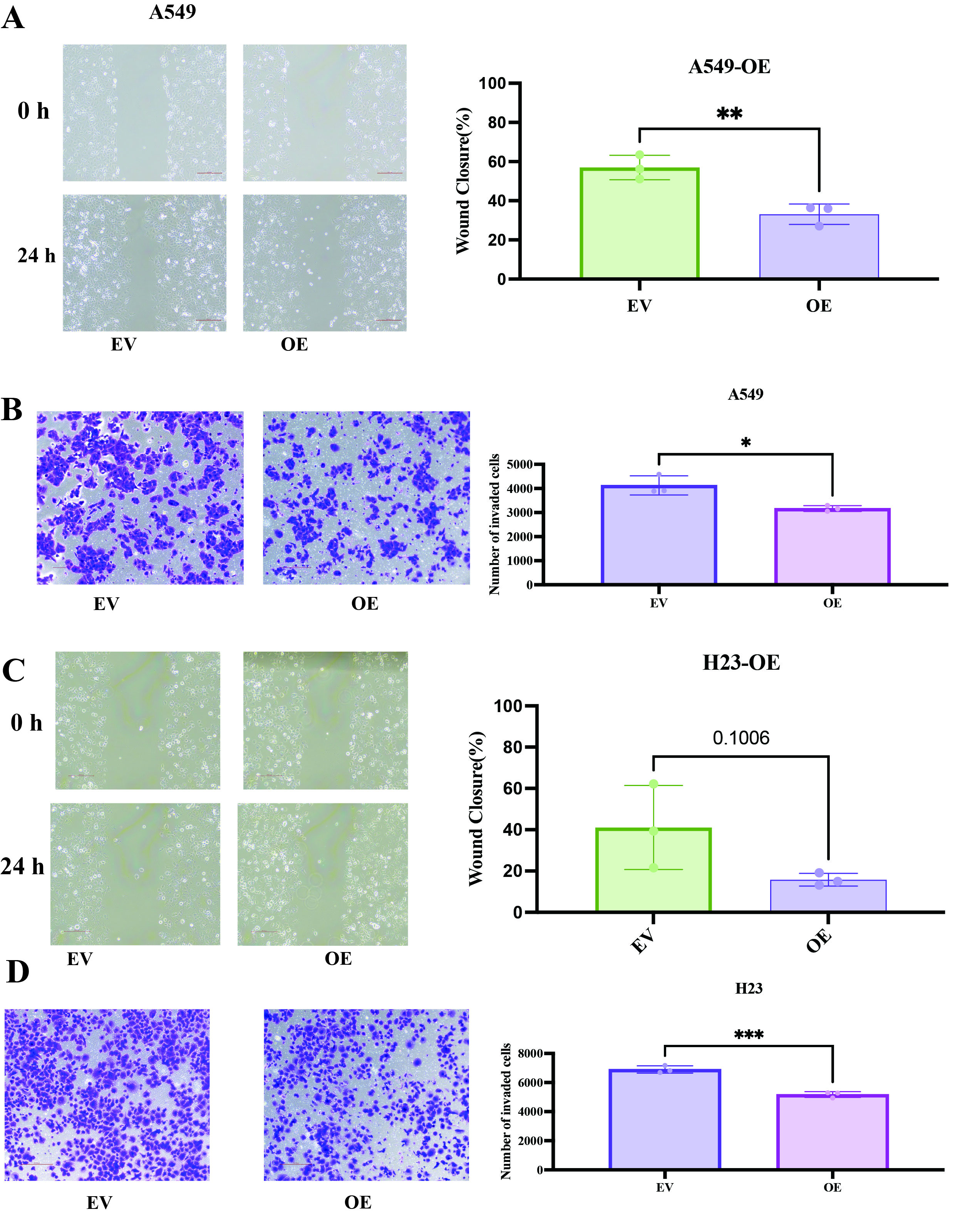

Supplement: Supplementary file 4 [file Image4.jpeg]
